# Changes in inflammation and oxidative stress signalling pathways in coarcted aorta triggered by bicuspid aortic valve and growth in young children

**DOI:** 10.3892/etm.2020.9171

**Published:** 2020-09-03

**Authors:** Katie L. Skeffington, Andrew R. Bond, M. Giulia Bigotti, Safa AbdulGhani, Dominga Iacobazzi, Sok-Leng Kang, Kate J. Heesom, Marieangela C. Wilson, Serban Stoica, Robin Martin, Massimo Caputo, M. Saadeh Suleiman, Mohamed T. Ghorbel

**Affiliations:** 1 Bristol Heart Institute, Research Floor Level 7, Bristol Royal Infirmary, Bristol BS2 8HW, UK; 2 Department of Congenital Heart Disease, Bristol Children's Hospital, Bristol BS2 8JB, UK; 3 Department of Physiology, Faculty of Medicine, Al-Quds University, P.O Box 89, Abu Dis, Palestine; 4 Proteomics Facility, University of Bristol, Bristol BS8 1RJ, UK

**Keywords:** aortic coarctation, bicuspid aortic valve, congenital heart disease

## Abstract

Neonates with coarctation of the aorta (CoA) combined with a bicuspid aortic valve (BAV) show significant structural differences compared to neonatal CoA patients with a normal tricuspid aortic valve (TAV). These effects are likely to change over time in response to growth. This study investigated proteomic differences between coarcted aortic tissue of BAV and TAV patients in children older than one month. Aortic tissue just proximal to the coarctation site was collected from 10 children (BAV; n=6, 1.9±1.7 years, TAV; n=4, 1.7±1.5 years, (mean ± SEM, P=0.92.) Tissue were snap frozen, proteins extracted, and the extracts used for proteomic and phosphoproteomic analysis using Tandem Mass Tag (TMT) analysis. A total of 1811 protein and 76 phosphoprotein accession numbers were detected, of which 40 proteins and 6 phosphoproteins were significantly differentially expressed between BAV and TAV patients. Several canonical pathways involved in inflammation demonstrated enriched protein expression, including acute phase response signalling, EIF2 signalling and macrophage production of IL12 and reactive oxygen species. Acute phase response signalling also demonstrated enriched phosphoprotein expression, as did Th17 activation. Other pathways with significantly enriched protein expression include degradation of superoxide radicals and several pathways involved in apoptosis. This work suggests that BAV CoA patients older than one month have an altered proteome consistent with changes in inflammation, apoptosis and oxidative stress compared to TAV CoA patients of the same age. There is no evidence of structural differences, suggesting the pathology associated with BAV evolves with age in paediatric CoA patients.

## Introduction

One of the most common congenital cardiac abnormalities is coarctation of the aorta (CoA) (1). Around 85% of CoA patients also have a bicuspid aortic valve (BAV) (2). Following CoA repair, patients are at increased risk of developing cardiovascular complications including hypertension, impaired left ventricular function, aortic aneurysms and aortic dissection (3-5). Several studies have demonstrated that the risks are greater in CoA patients with BAV compared to CoA patients with the normal tricuspid valve (TAV) (6-8). The co-existence of BAV and CoA is known to alter aortic blood flow haemodynamics, and this may underlie the increased susceptibility of CoA patients with BAV to cardiovascular complications (9,10).

A recent study by our group was the first to investigate differences in the vascular proteome of CoA patients with and without BAV (11). This study focused on neonatal patients (less than 3 weeks old) and demonstrated that the presence of BAV in neonatal CoA patients is associated with altered expression of proteins involved in elastin fibre formation and oxidative stress. In older CoA patients, there will have been more time for aortic remodelling to occur in response to the altered blood flow haemodynamics, and this will likely effect protein expression. Therefore, molecular changes in the coarctation area will be influenced not just by the valve and blood flow haemodynamics, but also the effects of growth. The current study therefore compares the proteome of CoA patients with and without BAV in paediatric patients older than one month, thus providing a unique insight into how the proteomic changes associated with BAV evolve in an older group of CoA patients.

## Materials and methods

### 

#### Patients and sample collection

Tissue was collected from paediatric patients older than one month undergoing congenital surgery including repair of an aortic coarctation. Tissue was collected just proximal to the coarctation site. The study was conducted in accordance with the declaration of Helsinki, and the protocol was approved by the North Somerset and South Bristol Research Ethics Committee (Health Research Authority, Whitefriars, Level 3 Block B, Lewins Mead, Bristol, BS1 2NT, REC 07/H0106/172). Full informed consent was obtained from parents prior to admission for operation. The tissue was snap frozen in liquid nitrogen before being stored at -80˚C (BAV: n=6, patient age 1.9±1.7 years (mean ± SEM). TAV: n=4, patient age 1.7±1.5 years).

#### Sample preparation

Proteins were extracted in radio-immuno-precipitation assay buffer (RIPA; 1% Nonidet P-40, 0.5% sodium deoxycholate, 0.1% SDS, in PBS with phosphatase and protease inhibitors), and quantified using Bradford's assay. Aliquots of 100 µg were digested (2.5 µg trypsin, 37˚C, overnight), labelled with Tandem Mass Tag (TMT) 10Plex reagents (Thermo Fisher Scientific, Loughborough, UK) and the labelled samples pooled. For total proteome analysis, aliquots of 50 µg of the pooled sample were dried, re-suspended in buffer A (20 mM ammonium hydroxide, pH 10) and fractionated by high pH reversed-phase (RP) chromatography (UltiMate 3000 liquid chromatography system (Thermo Fisher Scientific) with XBridge BEH C18 Column (130 Å, 3.5 µm, 2.1x150 mm, Waters, UK). The samples were loaded in buffer A and peptides eluted with an increasing gradient of buffer B (20 mM ammonium hydroxide in acetonitrile, pH 10, 0-95% over 60 min). The resulting fractions were dried and re-suspended in 1% (v/v) formic acid. The remainder of the TMT-labelled pooled sample was dried and enriched using a TiO_2_ based phosphopeptide enrichment protocol (Pierce), before further drying and re-suspension in 1% (v/v) formic acid.

#### Nano-LC mass spectrometry

Mass spectrometry was performed using an Ultimate 3000 nano-HPLC system in line with an Orbitrap Fusion Tribrid mass spectrometer (Thermo Scientific). The samples were injected onto an Acclaim PepMap C18 nano-trap column (Thermo Scientific), washed (0.5% v/v acetonitrile, 0.1% v/v formic acid) and resolved on a 250 mm x 75 µm Acclaim PepMap C18 reverse phase analytical column (Thermo Scientific) over an organic gradient (150 min, flow rate 300 nl/min, solvent A: 0.1% formic acid, solvent B: Aqueous 80% acetonitrile in 0.1% formic acid, seven gradient segments: 1-6% B over 1 min, 6-15% B over 58 min, 15-32% B over 58 min, 32-40% B over 5 min, 40-90% B over 1 min, held at 90% B for 6 min and then reduced to 1% B over 1 min). Nano-electrospray ionization was used to ionize the peptides (2.0 kV, stainless steel emitter internal diameter 30 µm (Thermo Scientific), capillary temperature 275˚C).

Spectra were acquired using an Orbitrap Fusion Tribrid mass spectrometer with Xcalibur 3.0 software (Thermo Scientific), operated using an SPS-MS3 workflow and data-dependent acquisition mode. For FTMS1 spectra a resolution of 120,000 was used, alongside an automatic gain control (AGC) target of 200,000 and a maximum injection time of 50 ms. Precursors were filtered with an intensity threshold of 5000, with monoisotopic peak determination set to peptide and to include charge states 2-7. Previously interrogated precursors were excluded with a dynamic window (60s +/-10 ppm). The MS2 precursors were isolated with a quadrupole mass filter (width of 1.2 m/z). ITMS2 spectra were collected (AGC target 10000, max injection time 70ms, CID collision energy 35%). FTMS3 analysis was then performed (resolution 50,000, AGC target 50,000, max injection time 105 ms). Fragmentation of precursors was achieved using high-energy collision dissociation at normalised collision energy of 60%. Synchronous Precursor Selection (SPS) was enabled to include up to five MS2 fragment ions in the FTMS3 scan.

#### Data processing and analysis

Processing and quantification of the raw data files was performed using Proteome Discoverer Software (Thermo Scientific, version 1.4). Peptide sequences were searched against the Uniprot human database (SEQUEST algorithm, peptide precursor mass tolerance 10 ppm, MS/MS tolerance 0.6Da). Oxidation of methionine (+15.9949) was included as a variable modification, and both carbamido-methylation of cysteine (+57.0214) and the addition of the TMT mass tag (+229.163) to peptide N-termini and lysine were included as fixed modifications. Phosphorylation of serine, threonine and tyrosine (+79.966) were also included as variable modifications in the phosphoproteomic analysis. Searches were performed with full tryptic digestion, allowing a maximum of one missed cleavage. The reverse database search option was enabled, and all the data filtered to satisfy false discovery rate of 5%.

Proteins or phosphoproteins with more than one missing value per group were excluded from the analysis (12), as were putative uncharacterized proteins and proteins with accession numbers representing cDNA with weak similarity. Values are presented as a ratio to the internal standard (a pool of all samples) and represent the median of the measured peptide(s) for each protein. Fold change (BAV/TAV) ratios were calculated, and log_2_ (fold-change) was plotted against -log_10_ (P-value) on a volcano plot. A change in protein expression greater than 1.3x or less than -1.3x and with P<0.05 (Student's t-test) was considered significant. These cut offs were chosen in accordance with other similar studies, for example (13). A heatmap was plotted using R software (version 3.5.1). QuickGo software was used to analyse gene ontology (GO) enrichment. Differentially expressed proteins and phosphoproteins were inputted into Ingenuity Pathway Analysis software (IPA version 46901286, Qiagen, Aarhus, Denmark) to determine significantly enriched canonical pathways (P-value of overlap calculated by Fisher's exact test right tailed).

## Results

The age range of patients in the study was from one month to 10.5 years. The average age was not different significantly between groups (TAV; 1.7±1.5 years, BAV; 1.9±1.7 years, P=0.92).

**Figure 1:**
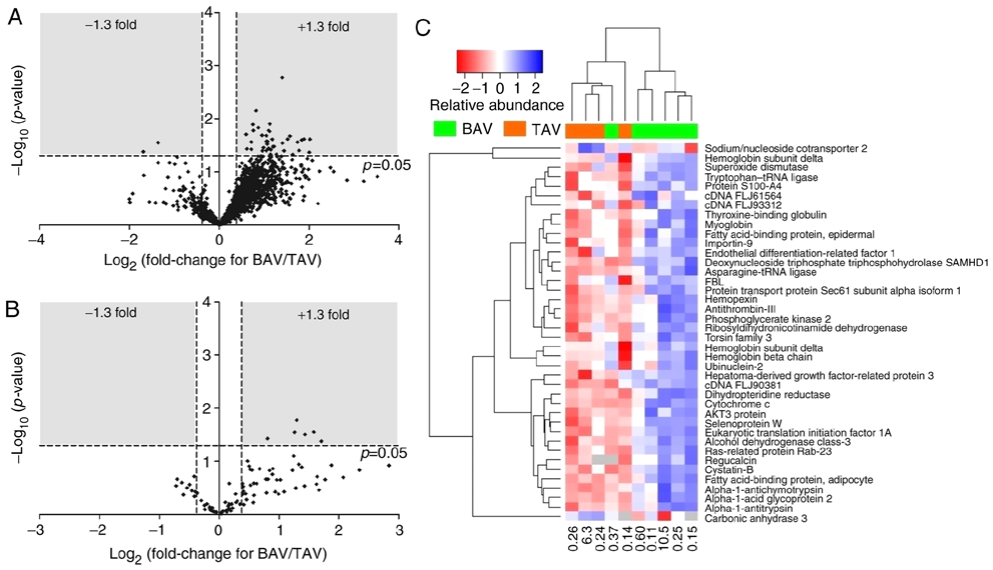
Differential protein expression. Volcano plots of proteins (A) and phosphoproteins (B) quantified in paediatric patients with bicuspid (BAV) vs. tricuspid (TAV) aortic valves. Each point represents the log_2_ (fold-change) between the two groups, plotted against the associated significance for the change. Proteins in the shaded area (>1.3 or <0.769 fold-change, P<0.05) are considered to be differentially expressed. (C) A heatmap of the 40 differentially expressed proteins. Horizontal dendrogram represents 10 independent samples. Vertical dendrogram represents the 40 proteins analysed, protein identities are listed on the right. The numbers at the bottom of each column represent the age of each patient, in years.

A total of 1811 protein accession numbers were detected, of which 40 were significantly differentially expressed between BAV and TAV patients (Fig. 1A and C, Table I); 38 proteins upregulated and two proteins downregulated in BAV patients compared to TAV patients. A total of 76 phosphorylated proteins were identified, some with multiple phosphorylation sites, resulting in 92 phosphorylation site matches. 6 phosphoproteins demonstrated significantly altered expression in BAV patients compared to TAV patients; all 6 demonstrated increased phosphorylation in BAV patients (Fig. 1B, Table II).

**Figure 2:**
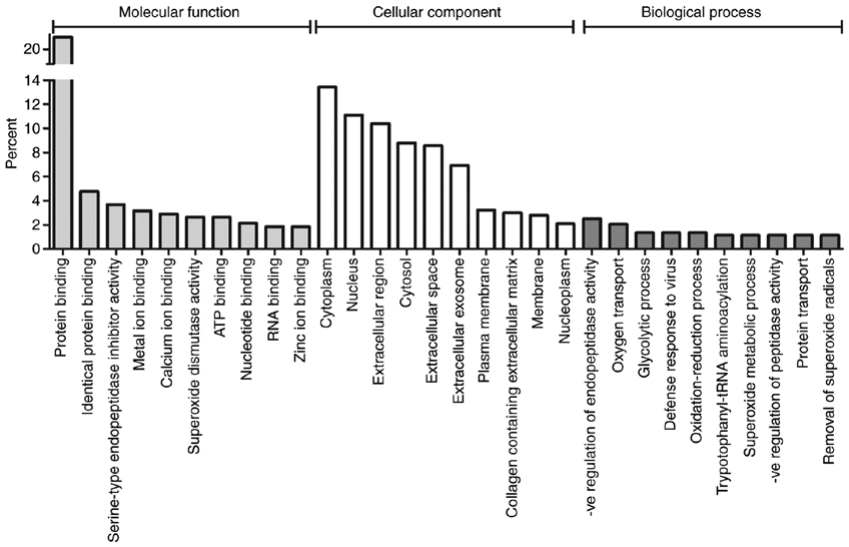
Functional classification by Gene Ontology (GO) analysis of proteins differentially expressed between BAV and TAV paediatric patients. The top 10 GO terms in each of the three main categories of GO classification (molecular function, cellular component and biological process) are displayed. The y axis represents the percentage of annotations per GO term.

Gene Ontology (GO) analysis was used to functionally classify the GO annotations associated with the differentially expressed proteins under the three main categories of GO analysis (molecular function, biological process and cellular component; Fig. 2). Numerous molecular functions relating to protein and ion binding were present in the top ten molecular functions, however endopeptidase and superoxide dismutase activities were also featured.

The top 20 significantly enhanced canonical pathways for protein expression are shown in Table III. The most significantly enhanced pathway was the acute phase response, a systemic response to inflammation. IPA analysis predicted activation of the acute phase response in BAV patients (z-score=2). Several other of the top 20 canonical pathways also relate to inflammation, including EIF2 signalling and macrophage production of IL12 and reactive oxygen species (ROS). Superoxide radical degradation was also highlighted as a significantly enriched canonical pathway. Finally, a number of canonical pathways involved in apoptosis, including Myc mediated apoptosis signalling, ceramide signalling and lymphotoxin β receptor signalling, were all significantly enriched. Sixteen canonical pathways were found to be significantly enriched from the phosphoproteomic data (Table IV), including two pathways relating to inflammation, namely the acute phase response and Th17 activation.

## Discussion

Our previous study (11) demonstrated that aortic tissue from neonatal CoA patients with BAV have proteomic and histological differences compared to TAV CoA patients of the same age. The changes included increased elastin content and altered expression of genes involved in elastin formation, inositol signalling and oxidative stress. The current study demonstrates that in older children (>one month) with CoA, there are still significant differences between the proteomes of BAV and TAV patients, but different proteins and pathways are involved, suggesting that the pathology associated with CoA not only develops with age, but also develops differently in BAV patients compared to TAV patients. Each group of patients contains one patient older than one year, whilst the others are aged between 1 month and one year. Interestingly, the protein expression profile of the two oldest patients are not the most extreme in either group (see Fig. 1C); this may suggest that the alterations in protein expression are similar in children between one month and ten years of age.

### 

#### The presence of BAV affects the expression of proteins involved in inflammatory pathways

The most significantly enriched proteomic canonical pathway was acute phase response signalling, which was predicted to be activated in BAV patients. This pathway was also significantly enriched in the phosphoproteomics data. The acute phase response is a systemic response triggered by major local inflammation and cytokine release. Several other inflammatory canonical pathways were also significantly enriched. Within the proteomic data, production of NO, ROS and the pro-inflammatory cytokine IL-12 by macrophages were enriched, as was EIF2 signalling [a pathway involved in the regulation of pro-inflammatory cytokine expression (14)]. In the phosphoproteome analysis, the third most significant canonical pathway was activation of Th17 cells, a subset of pro-inflammatory T helper cells (15).

These changes in canonical pathways related to inflammation are driven by significant upregulation of the abundance of several proteins and phosphoproteins involved in inflammation in BAV patients compared to TAV patients. α-1-antitrypsin (AAT), α-1-antichymotrypsin (ACT) and α-1-acid glycoprotein 2 (AGP) were all overexpressed in BAV patients compared to TAV patients. These proteins are all known to regulate macrophage function (16-18) and are all positive acute phase proteins (19,20) (proteins whose expression increases during an inflammatory episode), as is hemopexin (21), another protein overexpressed in BAV patients. AAT and ACT both belong to the serpin family of protease inhibitors and provide negative feedback of the acute phase response via their inhibition of inflammatory cells including neutrophils and mast cells (22,23). Another member of the serpin family with marked anti-inflammatory properties is anti-thrombin III (24), and this was also overexpressed in BAV patients. BAV patients also had overexpression of phosphorylated α-2 Heremans-Schmid glycoprotein [AHSG; a negative acute phase protein (25,26)] and phosphorylated HSP90AB1 [a member of the heat shock protein 90 family, which have been demonstrated to increase secretion of pro-inflammatory cytokines (27)].

Several studies have previously suggested that differences in basal inflammation may exist between BAV and TAV patients. Local inflammation is known to occur around the abnormal BAV valve (28), however whether the presence of BAV has an effect on aortic or systemic inflammation is less clear. The combination of CoA and BAV has been demonstrated to cause changes in aortic blood flow haemodynamics including altered indices of shear stress (9), factors which are, in turn, known to affect vascular inflammatory pathways (29). However, studies comparing inflammatory markers such as MMPs, myeloperoxidase or measures of macrophage infiltration in BAV vs. TAV patients have mixed conclusions: Some studies found an increase in inflammatory markers associated with BAV whilst others found no difference or reduced inflammation (30-34).

The trauma of cardiac surgery is known to activate the complement system even when no cardiopulmonary bypass is involved (35). Although the full immunological response takes hours to days to materialise, markers of complement activation and some markers of inflammation start increasing measurably during surgery itself (35), and therefore some inflammatory proteomic changes are likely to already be occurring at the timepoint our samples were taken. It is possible that the differences between BAV and TAV patients in the current study indicate that BAV patients have a greater inflammatory response to surgery than TAV patients. Possibly changes in both basal immunological status and in the response to surgery exist. One recent paper found that BAV patients have decreased T and B lymphocyte levels and the authors suggested that BAV patients have ‘an old immune system’ which is ‘more easily vulnerable to internal and external stressors’ (31).

#### The presence of BAV affects the expression of proteins involved in oxidative stress and apoptosis

The antioxidant enzyme cytosolic superoxide dismutase (SOD1) was significantly overexpressed in BAV CoA patients compared to CoA patients with a normal aortic valve, and superoxide radical degradation and superoxide dismutase activity were highlighted in canonical pathway analysis and gene ontology analysis respectively (Fig. 2 and Table III). These findings suggest there may be differences in the levels of oxidative stress between BAV and TAV CoA patients. Previous studies have demonstrated that adult BAV patients have increased aortic oxidative stress compared to similar patients with a morphologically normal aortic valve (36). This may be the result of altered mechanical stretch in the aortic walls of BAV patients as vascular mechanical stretch is a known stimulant of superoxide production (37). A previous study in adults found increased SOD1 expression in the aorta of some subsets of BAV patients (38); to the best of our knowledge the data in the current study is the first to suggest that an association of increased SOD1 expression with BAV also exists in paediatric CoA patients. However it is interesting that in our neonatal data set (11) there is no significant difference in SOD1 expression, suggesting the change in expression may only occur in older children.

Previous studies in neonates with BAV associated with CoA and adults with BAV have demonstrated decreased expression of the extracellular form of superoxide dismutase (SOD3) associated with BAV (11,38,39), however there was no significant change in SOD3 expression in the current data set. One of only two proteins to be significantly downregulated in BAV patients was however carbonic anhydrase III, which is known to have an antioxidant role under conditions of oxidative stress (40). Clearly much more work is required to understand the changes in oxidative stress and antioxidant defence mechanisms which occur at different ages in BAV patients with and without CoA.

Two proteins involved in apoptotic pathways, AKT3 and cytochrome c, were also significantly overexpressed in BAV CoA patients compared to TAV CoA patients. This resulted in the enrichment of a number of canonical pathways (Myc mediated apoptosis signalling, ceramide signalling and lymphotoxin β receptor signalling) which are involved in apoptosis (41-43). Several previous studies have reported an increase in vascular smooth muscle cell (VSMC) apoptosis in BAV patients, and have linked this to the increased incidence of in aortic dilation and aortic aneurysms in BAV patients (44-47). The degree to which this is a genetic effect or secondary to changes in blood flow haemodynamics, or a combination of both factors, is controversial (44,48-50).

Interestingly, there were also alterations in the ratio of haemoglobin chains. Various haemoglobin subunits are expressed in a number of non-erythroid cells, including lungs, neurons, endometrium and blood vessel walls (51,52). In the current study there was overexpression of haemoglobins beta and delta in the aorta of BAV CoA patients compared to TAV CoA patients. The role of the beta and delta subunits in the aortic wall is unclear however it has been suggested that haemoglobin α acts from within blood vessel walls to help regulate nitric oxide release and vascular tone (51). It is possible therefore that the observed changes relate to the altered blood flow haemodynamics in BAV patients, however much more work is needed to understand this.

Paediatric CoA patients with BAV have a greater long-term risk of cardiovascular complications than similar patients with TAV (6-8). This study demonstrates significant differences between the proteome of the coarcted aortic tissue of young CoA patients with and without BAV, indicating that inflammation, oxidative stress and apoptotic pathways could contribute to future complications associated with BAV patients. The structural changes observed in our previous study in neonates are no longer apparent. In the long-term, improved understanding of the molecular differences between these patients coupled with an understanding of how the changes evolve with age should help to optimise treatment strategies.

Limitations of the study include the lack of tissue from a healthy control group; this would help to elucidate how the proteomic differences observed in BAV patients in this study are affected by the presence of the CoA. However, such tissues would be very difficult to obtain. Using tissues from only diseased individuals may also increase the likelihood that pathways are involved which deviate from the canonical paradigms, and these may not have been fully uncovered by our analysis. Slight regional differences in the exact location of the tissue collection may also have introduced some variation into our analyses. Additionally, validation of the changes in protein expression using western blotting would be informative, however very small sample sizes made this difficult.

## Figures and Tables

**Table I:** Proteins differentially (fold increase >1.3 or <0.769) and significantly (P<0.05) expressed in coarcted aorta from paediatric BAV patients compared to paediatric TAV patients. - : gene ID not applicable.

			Mean		SEM					
Accession number	Gene ID	Description	BAV	TAV	BAV	TAV	Fold change (BAV/TAV)	P-value	log_2_ (fold-change)	-log_10_ (P-value)
P15090	FABP4	Fatty acid-binding protein, adipocyte	1.51	0.37	0.33	0.04	4.05	0.024	2.02	1.62
P19652	ORM2	α-1-acid glycoprotein 2	1.45	0.36	0.36	0.02	4.01	0.043	2.00	1.37
A0A024R943	TOR3A	Torsin family 3, member A, isoform CRA_b	2.04	0.55	0.42	0.14	3.72	0.024	1.89	1.62
Q6VFQ6	HBB	Hemoglobin beta chain (Fragment)	2.53	0.71	0.52	0.19	3.58	0.025	1.84	1.60
C9J6Y5	UBN2	Ubinuclein-2 (Fragment)	3.78	1.10	0.84	0.35	3.43	0.039	1.78	1.41
P01009	SERPINA1	α-1-antitrypsin	1.23	0.37	0.25	0.05	3.34	0.028	1.74	1.56
Q15493	RGN	Regucalcin	1.14	0.37	0.23	0.07	3.10	0.047	1.63	1.33
P02042	HBD	Hemoglobin subunit delta	3.13	1.05	0.55	0.30	2.98	0.021	1.57	1.67
P02790	HPX	Hemopexin	2.03	0.71	0.38	0.09	2.87	0.024	1.52	1.62
P01011	SERPINA3	α-1-antichymotrypsin	1.31	0.48	0.27	0.07	2.72	0.040	1.44	1.40
Q9ULC3	RAB23	Ras-related protein Rab-23	1.29	0.49	0.23	0.10	2.66	0.029	1.41	1.54
E9PEW8	HBD	Hemoglobin subunit delta (Fragment)	7.99	3.02	0.72	0.73	2.65	0.002	1.41	2.78
P01008	SERPINC1	Antithrombin-III	1.65	0.69	0.33	0.06	2.41	0.047	1.27	1.33
P04080	CSTB	Cystatin-B	0.96	0.41	0.17	0.06	2.32	0.033	1.22	1.48
P09417	QDPR	Dihydropteridine reductase	1.36	0.60	0.24	0.06	2.29	0.034	1.19	1.47
Q56A86	AKT3	AKT3 protein (Fragment)	1.21	0.54	0.19	0.06	2.26	0.024	1.18	1.61
B2R773	-	cDNA, FLJ93312, highly similar to Homo sapiens adipose most abundant gene transcript 1 (ADIPOQ), mRNA	1.92	0.87	0.32	0.20	2.22	0.038	1.15	1.42
Q5TD07	NQO2	Ribosyldihydronicotinamide dehydrogenase [quinone]	1.54	0.70	0.25	0.21	2.20	0.047	1.14	1.33
Q96P70	IPO9	Importin-9	1.53	0.70	0.19	0.14	2.18	0.013	1.12	1.90
O14602	EIF1AY	Eukaryotic translation initiation factor 1A, Y-chromosomal	1.18	0.55	0.16	0.08	2.17	0.017	1.12	1.76
A0A024R0V8	SEPW1	Selenoprotein W, 1 (Fragment)	1.37	0.64	0.18	0.11	2.14	0.017	1.10	1.78
P05543	SERPINA7	Thyroxine-binding globulin	1.79	0.84	0.29	0.14	2.14	0.036	1.10	1.44
B4E367	-	cDNA FLJ61564, highly similar to Plexin domain-containing protein 2	2.18	1.03	0.33	0.13	2.11	0.027	1.08	1.57
B3KQF5	-	cDNA FLJ90381 fis, clone NT2RP2005035, highly similar to Calumenin	0.96	0.46	0.14	0.09	2.09	0.030	1.07	1.53
C9JFR7	CYCS	Cytochrome c (Fragment)	1.28	0.62	0.19	0.02	2.08	0.027	1.05	1.57
P11766	ADH5	Alcohol dehydrogenase class-3	1.12	0.54	0.15	0.07	2.06	0.021	1.04	1.69
P07205	PGK2	Phosphoglycerate kinase 2	1.52	0.74	0.25	0.09	2.06	0.040	1.04	1.40
Q01469	FABP5	Fatty acid-binding protein, epidermal	1.75	0.88	0.26	0.14	1.98	0.035	0.99	1.46
Q9Y3E1	HDGFRP3	Hepatoma-derived growth factor-related protein 3	1.02	0.52	0.13	0.16	1.95	0.040	0.96	1.40
P02144	MB	Myoglobin	1.67	0.86	0.21	0.14	1.94	0.021	0.96	1.69
P26447	S100A4	Protein S100-A4	2.36	1.30	0.21	0.29	1.82	0.015	0.86	1.83
P61619	SEC61A1	Protein transport protein Sec61 subunit α isoform 1	1.16	0.66	0.11	0.06	1.75	0.007	0.81	2.15
Q96BS4	FBL	FBL protein (Fragment)	1.25	0.74	0.09	0.16	1.69	0.017	0.75	1.78
P23381	WARS	Tryptophan-tRNA ligase, cytoplasmic	1.74	1.04	0.15	0.15	1.68	0.013	0.74	1.89
O43776	NARS	Asparagine-tRNA ligase, cytoplasmic	1.18	0.72	0.15	0.05	1.64	0.037	0.71	1.43
Q9Y3Z3	SAMHD1	Deoxynucleoside triphosphate triphosphohydrolase SAMHD1	1.27	0.78	0.15	0.04	1.64	0.027	0.71	1.56
P00441	SOD1	Superoxide dismutase [Cu-Zn]	1.92	1.26	0.15	0.24	1.53	0.037	0.62	1.43
O60869	EDF1	Endothelial differentiation-related factor 1	1.30	0.86	0.07	0.16	1.51	0.020	0.60	1.71
P07451	CA3	Carbonic anhydrase 3	1.47	3.78	0.45	0.73	0.39	0.028	-1.37	1.55
O43868	SLC28A2	Sodium/nucleoside cotransporter 2	5.72	18.47	1.44	6.25	0.31	0.042	-1.69	1.38

**Table II:** Phosphoproteins differentially (fold increase >1.3 or <0.769) and significantly (P<0.05) expressed in coarcted aorta from paediatric BAV patients compared to paediatric TAV patients. - : gene ID not applicable.

				Mean		SEM					
Accession number	Gene ID	Description	Modification	BAV	TAV	BAV	TAV	Fold-change (BAV/TAV)	P-value	log_2_ (fold change)	-log_10_ (P-value)
B7Z556	-	cDNA FLJ56822, highly similar to α-2-HS-glycoprotein (AHSG)	S7(Phospho)	0.88	0.27	0.20	0.04	3.27	0.042	1.709	1.381
K7EKZ3	VPS4B	Vacuolar protein sorting-associated protein 4B	S4(Phospho)	2.02	0.68	0.39	0.18	2.98	0.028	1.577	1.550
Q6PK50	HSP90AB1	HSP90AB1 protein (Fragment)	S6(Phospho)	0.95	0.35	0.17	0.11	2.70	0.031	1.433	1.504
Q09666	AHNAK	Neuroblast differentiation-associated protein AHNAK	S8(Phospho)	1.05	0.43	0.15	0.11	2.45	0.017	1.292	1.777
Q53TN4	CYBRD1	Cytochrome b reductase 1	S2(Phospho)	1.88	0.79	0.31	0.18	2.39	0.028	1.255	1.547
B1AKZ5	PEA15	Astrocytic phosphoprotein PEA-15	S3(Phospho)	1.30	0.74	0.14	0.17	1.75	0.037	0.807	1.428

**Table III:** Top twenty significant canonical pathways with enriched protein expression. IPA: Ingenuity Pathway Analysis.

IPA canonical pathway	P-value	Molecule(s)
Acute Phase Response Signalling	1.52x10^-5^	HPX, AKT3, ORM2, SERPINA1, SERPINA3
FXR/RXR Activation	7.10x10^-5^	HPX, AKT3, ORM2, SERPINA1
Amyotrophic Lateral Sclerosis Signalling	1.04x10^-3^	AKT3, CYCS, SOD1
LXR/RXR Activation	1.27x10^-3^	HPX, ORM2, SERPINA1
Coagulation System	1.74x10^-3^	SERPINC1, SERPINA1
Iron homeostasis signalling pathway	1.81x10^-3^	HPX, HBD, HBB
tRNA Charging	2.16x10^-3^	NARS, WARS
IL-12 Signalling and Production in Macrophages	2.26x10^-3^	ORM2, AKT3, SERPINA1
Formaldehyde Oxidation II (Glutathione-dependent)	3.53x10^-3^	ADH5
Docosahexaenoic Acid (DHA) Signalling	4.10x10^-3^	AKT3, CYCS
Production of Nitric Oxide and Reactive Oxygen Species in Macrophages	5.55x10^-3^	ORM2, AKT3, SERPINA1
Lymphotoxin β Receptor Signalling	6.61x10^-3^	AKT3, CYCS
Phenylalanine Degradation I (Aerobic)	7.04x10^-3^	QDPR
EIF2 Signalling	7.90x10^-3^	WARS, AKT3, EIF1AY
Myc Mediated Apoptosis Signalling	8.79x10^-3^	AKT3, CYCS
Small Cell Lung Cancer Signalling	1.03x10^-2^	AKT3, CYCS
Superoxide Radicals Degradation	1.40x10^-2^	SOD1
Ceramide Signalling	1.45x10^-2^	AKT3, CYCS
VEGF Signalling	1.73x10^-2^	AKT3, EIF1AY
Glucose and Glucose-1-phosphate Degradation	1.92x10^-2^	RGN

**Table IV:** Significant canonical pathways with enriched phosphoprotein expression. IPA: Ingenuity Pathway Analysis.

IPA canonical pathway	P-value	Molecule
Mitotic Roles of Polo-Like Kinase	1.77x10^-2^	HSP90AB1
Hypoxia Signalling in the Cardiovascular System	1.99x10^-2^	HSP90AB1
Th17 Activation Pathway	2.44x10^-2^	HSP90AB1
Prostate Cancer Signalling	2.47x10^-2^	HSP90AB1
Neuregulin Signalling	2.57x10^-2^	HSP90AB1
Nitric Oxide Signalling in the Cardiovascular System	2.68x10^-2^	HSP90AB1
PPAR Signalling	2.78x10^-2^	HSP90AB1
Telomerase Signalling	2.89x10^-2^	HSP90AB1
LXR/RXR Activation	3.23x10^-2^	AHSG
FXR/RXR Activation	3.36x10^-2^	AHSG
PI3K/AKT Signalling	3.52x10^-2^	HSP90AB1
Iron homeostasis signalling pathway	3.65x10^-2^	CYBRD1
Aryl Hydrocarbon Receptor Signalling	3.81x10^-2^	HSP90AB1
Aldosterone Signalling in Epithelial Cells	4.23x10^-2^	HSP90AB1
eNOS Signalling	4.26x10^-2^	HSP90AB1
Acute Phase Response Signalling	4.75x10^-2^	AHSG

## Data Availability

The datasets used and/or analyzed during the current study are available from the corresponding author on reasonable request.
